# Deciphering the Multi-Chromosomal Mitochondrial Genome of *Populus simonii*

**DOI:** 10.3389/fpls.2022.914635

**Published:** 2022-06-15

**Authors:** Changwei Bi, Yanshu Qu, Jing Hou, Kai Wu, Ning Ye, Tongming Yin

**Affiliations:** ^1^Key Laboratory of Tree Genetics and Biotechnology of Educational Department of China, Key Laboratory of Tree Genetics and Sivilcultural Sciences of Jiangsu Province, College of Forestry, Nanjing Forestry University, Nanjing, China; ^2^College of Information Science and Technology, Nanjing Forestry University, Nanjing, China

**Keywords:** *Populus simonii*, mitochondrial genome, fission, multi-circular molecule, comparative analysis

## Abstract

Mitochondria, inherited maternally, are energy metabolism organelles that generate most of the chemical energy needed to power cellular various biochemical reactions. Deciphering mitochondrial genome (mitogenome) is important for elucidating vital activities of species. The complete chloroplast (cp) and nuclear genome sequences of *Populus simonii* (*P. simonii*) have been reported, but there has been little progress in its mitogenome. Here, we assemble the complete *P. simonii* mitogenome into three circular-mapping molecules (lengths 312.5, 283, and 186 kb) with the total length of 781.5 kb. All three molecules of the *P. simonii* mitogenome had protein-coding capability. Whole-genome alignment analyses of four *Populus* species revealed the fission of poplar mitogenome in *P. simonii*. Comparative repeat analyses of four *Populus* mitogenomes showed that there were no repeats longer than 350 bp in *Populus* mitogenomes, contributing to the stability of genome sizes and gene contents in the genus *Populus*. As the first reported multi-circular mitogenome in *Populus*, this study of *P. simonii* mitogenome are imperative for better elucidating their biological functions, replication and recombination mechanisms, and their unique evolutionary trajectories in *Populus*.

## Introduction

Mitochondria are membrane-bound organelles only present in the cytoplasm of most eukaryotic (plants, animals, and fungi) cells. The primary function of mitochondria is to generate Adenosine triphosphate (ATP) by oxidative phosphorylation and produce metabolic intermediates for various cellular processes, hence it is popularly known as the “powerhouse” or “energy factory” of the cell (Krömer et al., 1988; Klingenberg, 2008). The outer mitochondrial membrane serves as a signal transmission platform, providing a scaffold for many critical proteins involved in cellular signaling (Liu et al., 1996; Mignotte and Vayssiere, 1998). Plant mitochondria are signaling organelles that participate in multiple biological processes, including programmed cell death, proliferation, respiration, and metabolic adaptation (similar to animal mitochondria) (Mignotte and Vayssiere, 1998; Chandel, 2021). Additionally, they also participate in conferring male sterility (Liberatore et al., 2016; Kim and Zhang, 2018; Siqueira et al., 2018). Plant mitochondria are well-known for their extreme variation in genome size, mutation rates, structural complexity, and ability to incorporate foreign DNA (Petersen et al., 2020). With the rapid development of next-generation sequencing (NGS) and third-generation sequencing (TGS) technologies, especially the emergence of Pacific Biosciences (PacBio) and Oxford Nanopore, an increasing number of plant organelle genomes have been assembled and submitted to NCBI GenBank. Up to February 2022, over 6800 complete plant chloroplast and plastid genomes have been deposited in GenBank Organelle Genome Resources^1^, but only 437 plant mitogenomes have been assembled. Among the assembled plant mitogenomes, 229 (more than half in total) were submitted in the past 5 years (2017–2021). The huge difference in the numbers of released mitochondrial and chloroplast genomes indicates that plant mitogenome is very complicated and difficult to assemble (Bi et al., 2020).

Plant mitogenomes are conventionally depicted as circular molecules, much like the circular chromosomes in animal mitochondria and bacteria (Kozik et al., 2019). However, the increased availability of plant mitogenomes have revealed that the *in vivo* structure of plant mitogenome is far more complex than a single circular chromosome model would suggest (Sloan, 2013). For example, the cucumber (*Cucumis sativus*) mitogenome was assembled into one large circular chromosome (1556 kb) and two small circular chromosomes (45 and 84 kb), of which only the large chromosome has protein-coding capability (Alverson et al., 2011). The copy number of the large chromosome is approximately twice as abundant as the two small chromosomes, suggesting the independent replication of the three mt chromosomes in cucumber plant cells (Alverson et al., 2011; Wu Z. Q. et al., 2020). Additionally, the *Amborella trichopoda* (*A. trichopoda*) mitogenome was found to have five circular chromosomes with lengths ranging from 119 to 3179 kb (Rice et al., 2013). For the holoparasitic plant, *Lophophytum mirabile*, the whole mitogenome (822 kb) was divided into 54 separate circular chromosomes with lengths ranging from 7.2 to 580 kb (Sanchez-Puerta et al., 2017). The largest and most complex multi-chromosomal mitogenomes have been found in the genus *Silene*, whose mitogenomes are often very large (>6000 kb) and contain a large number of circular chromosomes (Sloan et al., 2012; Wu et al., 2015a,b). However, dozens of the “empty” chromosomes in the *Silene* multi-chromosomal mitogenomes exhibit no protein-coding capability. Recently, the mitogenome of another holoparasitic plant, *Rhopalocnemis phalloides*, has been reported to consist of 21 minicircular chromosomes ranging from 4.95 to 7.86 kb with a shared region containing the replication origin, and replicates via a rolling circle mechanism (Yu et al., 2022). With extensive sampling of *Silene noctiflora* from 24 different populations, Wu and Sloan predicted that the dominating pattern of the multi-chromosomal variations should be the chromosome loss events after large ancestral expansions (Wu and Sloan, 2019). However, they could not get the conclusive result only from the limited genomic data. The multi-chromosomal mitogenome have also been found in many other species, but none was reported in genus *Populus*. Up to the present, only four *Populus* mitogenomes have been released, including *Populus tremula* (Kersten et al., 2016), *P. davidiana* (Choi et al., 2017), *P. alba* (Brenner et al., 2019), and *P. tremula* × *P. alba* (Kersten et al., 2016), all of which were assembled into the typical single circular structure. Accurate characterizations of plant mitogenome structures are imperative for better elucidating their biological functions, replication and recombination mechanisms, and their unique evolutionary trajectories (Kozik et al., 2019).

*Populus simonii* Carrière, commonly known as Chinese Cottonwood, is a fast-growing deciduous tree with shiny green, diamond-shaped leaves, mainly distributed from Qinghai to the east coast and from the Heilongjiang River to the Yangtze River (Wei et al., 2012). It is a major industrial tree species for building, urban landscaping, pulping, furniture, ecological protection, and biofuels (Yang et al., 2021). Chinese Cottonwood is also a primary tree species in preventing desertification, reducing soil erosion, counteracting wind damage, and fixing sand dunes in Northeast, North and Northwest China. Additionally, considering its drought resistance, barren tolerance, wide adaptability, strong rooting ability and interspecific cross-compatibility, *P. simonii* has been regarded as one of the best parents for breeding poplar clone varieties (Wu H. et al., 2020). Recently, the complete chloroplast (NC_037418.1) and nuclear (GCA_007827005.2) genome sequences of *P. simonii* have been released (Wu H. et al., 2020), but its mitogenome sequence is currently not available, hindering the development of new varieties with wider adaptive and commercial traits. In this study, we assembled the complete multi-circular *P. simonii* mitogenome based on the WGS data from the PacBio Sequel platform. Each chromosome of the *P. simonii* mitogenome has protein-coding capability. As the first reported multi-circular mitogenome in *Populus*, this study of *P. simonii* mitogenome will not only provide an important genetic resource for the comparative and functional genomic research in *Populus*, but also furnish an effective assembly strategy for other plant species, especially for species with difficulties in assembly.

## Materials and Methods

### Plant Material, DNA Extraction and Genome Sequencing

The male *P. simonii* seeds provided by Luoning Bureau of Forest, Henan Province, China and planted at the Xiashu Forest Farm of Nanjing Forestry University, Jurong, Jiangsu Province, China. Fresh leaves were collected and immediately frozen in liquid nitrogen, followed by preserving the leaves at –80°C in the laboratory prior to DNA extraction. The total genomic DNA was extracted from leaves using the CTAB protocol (Arseneau et al., 2017), and a library with an insert size of 20 kb was constructed using a BluePippin DNA size selection instrument (Sage Science, Beverly, MA, United States) with a lower size limit of 10 kb. The prepared library was sequenced on the PacBio Sequel platform (Pacific Biosciences, United States) at Frasergen Technologies Corporation, Wuhan, China. After the removal of adapter sequences using SMRTlink (v7.0.1^2^), the remaining sequences were used for genome assembly and error correction. Additionally, to collect the contigs assembled by the PacBio sequencing data, we also sequenced the same genomic DNA afore-described on the Illumina HiSeq 2000 sequencing platform at Biomarker Technologies Corporation, Beijing, China.

### Mitochondrial Genome Assembly

**FIGURE 1:**
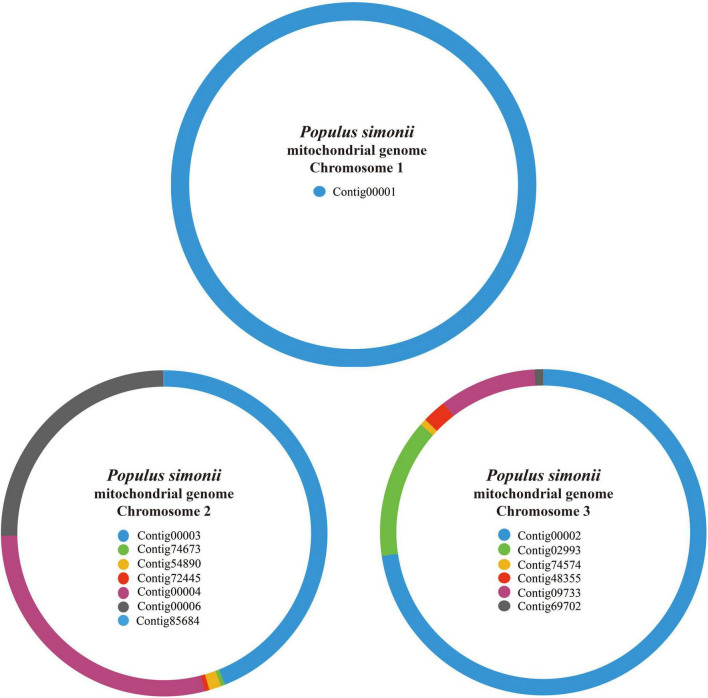
The assembly graph of *P. simonii* mitogenome. The mt contigs chosen from Newbler are represented by different color blocks. The *P. simonii* mtChr1 is composed of only one contig, while mtChr2 and mt Chr3 are composed of seven and six contigs, respectively.

PacBio Sequel and Illumina HiSeq 2000 hybrid strategies were applied to assemble the whole mitogenome of *P. simonii*. The PacBio long reads were used to *de novo* assemble the draft mitogenome, and the Illumina short reads were used to polish the draft mitogenome. Since the raw PacBio reads contain high systematic error rate, Canu v2.1.1 was used to correct the raw sequencing data prior to the initial assembly (Koren et al., 2017). The error-corrected long reads were then fed to Newbler v3.0 assembler for de novo assembling the *P. simonii* mitogenome. Since Newbler v3.0 can only assemble sequencing reads with a length less than 30 kb, the overlong reads (>30 kb) were first disconnected into two or more shorter reads using local Perl scripts. Then, Newbler was used to assemble the preprocessed reads with the following parameters: -cpu 20, -het, -sio, -m, -urt, -large, and -s 100 (Bi et al., 2016; Ye et al., 2017). Next, BlastN was applied to extract the potential mitochondrial contigs using other three *Populus* mitogenomes (*P. alba*, *P. davidiana*, and *P. tremula*) as references (Altschul et al., 1990), and all potential mitochondrial contigs were then confirmed based on their read depths (Supplementary Figure S1). Using Perl scripts^3^, the potential mitochondrial contigs were connected to construct the draft assembly graph based on the file “454ContigGraph.txt,” which generated from Newbler v3.0 and recorded all the relatedness of contig connections (Wang et al., 2011; Zhang et al., 2011). According to read depths of these connected contigs, some false links and forks were manually removed, and finally 14 contigs were obtained to assemble the *P. simonii* mitogenome (Figure 1).

### Mitogenome Verification and Correction

To ensure the correct assembly of the three chromosomes in *P. simonii* mitogenome, another de novo assembler SmartDenovo was used to assemble the *P. simonii* mitogenome (Liu et al., 2021). Mitochondrial contigs were identified by searching the assembled contigs against the three chromosomes generated from Newbler using BlastN. The aligned results showed that three contigs (utg1588, utg1107, and utg5276) belonged to the *P. simonii* mitogenome. All the three contigs were found to be self-assembled into a single circular chromosome by searching themselves using BlastN, which validates the multi-circular mitogenome of *P. simonii*. Additionally, we used BlastN to acquire the break sites between the *P. tremula* mitogenome and three *P. simonii* mitochondrial chromosomes, and then extracted the sequences of 500 bp on both sides of the break sites. Then, BlastN was used to aligned the error-corrected PacBio reads to the break sites (500 bp on both sides) to determine the multi-circular mitogenome of *P. simonii*.

To improve the accuracy of the mitogenome assembly, the draft mitogenome was polished with both PacBio long reads and Illumina short reads. For PacBio long reads correcting, all the PacBio clean reads were mapped onto the draft mitogenome using minimap2 (Li, 2018), and then the errors were corrected using racon^4^ with default parameters. For Illumina short reads correction, BWA mem (v0.7.17) (Li and Durbin, 2009) was used to map all the pair-end (PE) reads of the same individual onto the mitogenome of *P. simonii* that generated from correcting processes of the PacBio long reads. Then, SAMtools (v1.11) (Danecek et al., 2021) was used to convert the output files to BAM format, and Pilon (v1.23) (Walker et al., 2014) was applied to further correct the mitogenome with default parameters.

### Mitogenome Annotation

The public MITOFY analysis web server^5^ (Alverson et al., 2010) was employed to predict the protein-coding genes, tRNA and rRNA genes of the complete *P. simonii* mitogenome. The putative protein-coding genes (PCGs) were manually checked and adjusted by referring to other *Populus* mitogenomes, including *P. alba*, *P. tremula*, and *P. davidiana*. The tRNA and rRNA genes were confirmed using tRNAscan-SE v1.21 (Schattner et al., 2005) and RNAmmer 1.2 Server (Lagesen et al., 2007). Finally, the annotations of PCGs, tRNA and rRNA genes were integrated and manually reviewed using MacVector v18.0. The online program GeSeq^6^ were used to visualize the multi-circular genome map of *P. simonii* mitogenome (Tillich et al., 2017).

### Whole-Genome Alignment and Repeat Analyses in *Populus*

To verify the fission of *P. simonii* mitogenome from other *Populus* mitogenomes, the nucmer program of MUMmer v3.23 (Kurtz et al., 2004) was used to align the mitogenomes of *P. simonii*, *P. davidiana* (NC_035157.1), and *P. alba* (NC_041085.1) against the *P. tremula* mitogenome (NC_028096.1). Then, the delta-filter program of MUMmer was used to filter the alignments from nucmer with the minimum alignment identity of 80% and minimum alignment length of 100 bp. Mummerplot was used to visualize the alignment results produced by mummer and delta-filter by using the GNU gnuplot utility.

The simple sequence repeats (SSRs) of the *P. simonii* mitogenome were detected using the online Microsatellite identification tool^7^ with an SSR motif length of 1, 2, 3, 4, 5, and 6 bases and thresholds of 8, 4, 4, 3, 3, and 3 repeat numbers, respectively (Beier et al., 2017). The tandem repeats were detected using the online tool Tandem Repeats Finder v4.09^8^ with default parameters (Benson, 1999). The dispersed repeats were detected using online REPuter program^9^ with the minimal repeat size of 30 bp and hamming distance of 3 (Kurtz et al., 2001).

### Phylogenetic Analysis

To accurately infer the phylogenetic relationships of *P. simonii*, the conserved PCGs from the mitogenomes of *P. simonii* and 34 other plants were used to construct the phylogenetic tree. These observed mitogenomes were downloaded from NCBI, and the accession numbers and abbreviations were listed in Supplementary Table S1. A local Perl script was used to identify the conserved PCGs of all observed mitogenomes^10^. The selected 22 PCGs (*atp1*, *atp4*, *atp6*, *atp8*, *atp9*, *ccmB*, *ccmC*, *ccmFc*, *ccmFn*, *cob*, *cox1*, *cox2*, *cox3*, *nad1*, *nad2*, *nad3*, *nad4*, *nad4L*, *nad5*, *nad6*, *nad7*, and *nad9*) were concatenated into a single dataset of FASTA format, and then aligned using Muscle software with default settings (Edgar, 2004). Subsequently, IQ-TREE v2.1.4 (Minh et al., 2020) was used to construct the maximum likelihood-based phylogenetic tree with the following settings: -m MFP -B 1000 –bnni -T AUTO. The best evolutionary model was chosen as ‘GTR+F+R4’ according to the Bayesian Information Criterion (BIC) scores generated from IQ-TREE. The bootstrap value (%) in which the associated taxa clustered together was inferred from 1000 replications.

## Results

### Genome Assembly of the Multi-Circular Mitogenome of *Populus simonii*

For PacBio Sequel platform, a total of 604,419 PacBio reads representing ∼7.6 Gb were generated, with an average read length of 12,589 bp, and the longest read length was 112,390 bp (Supplementary Table S2). To correct the draft mitogenome generated from PacBio sequencing data, a total of 327 million PE reads representing 32.7 Gb were also generated using the Illumina HiSeq 2000 sequencing platform. After the overlong reads were cut into two or more shorter reads using local Perl scripts, 663,890 reads were obtained for subsequent assembly. The de novo assembly of Newbler generated 90,449 contigs, and the longest contig was 312,303 bp (Supplementary Table S2). After removing false links and some wrong forks from horizontal gene transfer (HGT), 14 contigs were obtained to construct the draft contig connection graph of *P. simonii* mitogenome (Figure 1). As shown in Table 1, the genome coverage of each mitochondrial contig was approximately 70×, and only Contig85684 (1036.3×) and Contig69702 (113.7×) showed some anomalies, of which the former belonged to cp-derived sequences and partial sequences of the latter belonged to dispersed repeats. The mitogenome was composed of three circular chromosomes, and the largest one (length: 312,303 bp) was assembled by only one contig (Contig00001), the second one (length: 282,737 bp) was assembled by seven contigs (Contig00003, Contig00004, Contig00006, Conti54890, Contig72445, Contig74673, and Contig85684), and the shortest one (length: 185,980 bp) was assembled by six contigs (Contig00002, Contig02993, Contig74574, Contig48355, Contig09733, and Contig69702).

**TABLE 1:** Newbler assembled contigs of *P. simonii* mitogenome.

Chromosome	Contig name	Coverage (×)	Number of reads	Length (bp)
mtChr1	Contig00001	73.3	1,931	312,303
mtChr2	Contig00003	70.4	757	124,150
	Contig00004	61.1	455	81,303
	Contig00006	71.3	444	71,063
	Contig54890	68.9	98	3,376
	Contig72445	88.1	109	1363
	Contig74673	73.3	79	1191
	Contig85684^a^	1036.2	1,059	291
mtChr3	Contig00002	72.6	927	135,137
	Contig02993	71.7	197	25,710
	Contig09733	65.7	144	17,871
	Contig48355	67	99	4,456
	Contig69702	113.7	123	1,607
	Contig74574	82.3	96	1199
Average/Total	14	75.3^b^	6,518	781,020

*^a^cp-derived.*

*^b^The average coverage of 13 mt contigs without one cp-derived contig.*

To validate the assembly of the multi-circular mitogenome of *P. simonii*, we used BlastN to acquire the break sites between the *P. simonii* and *P. tremula* mitogenomes (Supplementary Figure S2), and then mapped all the error-corrected PacBio reads to the break sites (500 bp on both sides). A total of 719 PacBio reads were found to be similar to the sequences around the break sites (identity > 95%, length > 450 bp), but almost none of them spanned more than 60% of the entire sequences (Supplementary Table S3), suggesting that the multi-circular mitogenome of *P. simonii* was assembled accurately. Subsequently, a more accurate multi-circular mitogenome was obtained after polishing with both PacBio long reads and Illumina short reads.

### Genomic Features of the *Populus simonii* Mitogenome

**FIGURE 2:**
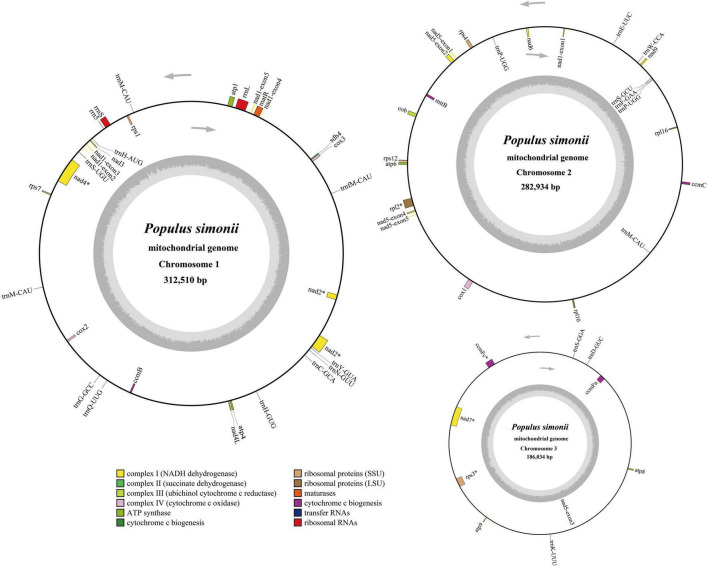
Circular maps of the multi-circular mitogenome of *P. simonii*. Genomic features on transcriptionally clockwise and counterclockwise strands are drawn on the inside and outside of the three circles, respectively. GC content of each chromosome is represented on the inner circle by the dark gray plot. The asterisks (*) besides genes denote intron-containing genes.

The multi-circular mitogenome of *P. simonii* has been submitted to NCBI Genome Database under the GenBank accessions: MZ905370, MZ905371, and MZ905372. The whole *P. simonii* mitogenome was assembled into three circular chromosomes, with an atypical multi-circular conformation rather than the typical single circular structure in most plant mitogenomes (Guo et al., 2017). The largest circular chromosome (mtChr1) was 312,510 bp in length, the second one (mtChr2) was 282,934 bp in length, and the shortest one (mtChr3) was 186,034 bp in length (Figure 2). The *P. simonii* mitogenome (Size: 781,478 bp, GC content: 44.78%) was similar in size and GC content to that of other *Populus* mitogenomes (Table 2), such as *P. tremula* (Size: 783.4 kb, GC content: 44.75%), *P. alba* (Size: 838.4 kb, GC content: 44.82%), and *P. davidiana* (Size: 781.5 kb, GC content: 44.82%).

**TABLE 2:** General mitogenomic features of four representative plants in the genus *Populus*.

Feature	*P. simonii*	*P. alba*	*P. davidiana*	*P. tremula*
Genome size (bp)	781,478	838,420	779,361	783,442
GC content (%)	44.78	44.82	44.82	44.75
No. of PCGs	33	34	34	34
No. of tRNA genes	21	22	22	22
No. of rRNA genes	3	3	3	3
No. of exons	30	30	30	30
No. of introns	17	17	17	17
Coding regions (bp)	64,483 (8.25%)	63,781 (7.61%)	64,099 (8.22%)	64,153 (8.19%)
tRNA	1,583	1,663	1,663	1,663
rRNA	5,365	5,369	5,369	5,373
PCGs	30,237	29,694	30,063	30,063
*Cis*-spliced introns (bp)	27,298	27,055	27,004	27,054
Intergenic regions (bp)	716,995 (91.75%)	774,639 (92.39%)	715,262 (91.78%)	719,289 (91.81%)

There are 57 genes annotated in the *P. simonii* mitogenome, including 33 protein-coding genes (PCGs), 21 tRNA genes and 3 rRNA genes (Table 2). Since this mitogenome did not have large repeated regions, all the annotated genes were single-copy genes, except for two tRNA genes (*trnM-CAU* and *trnP-UGG*). The total length of coding sequences (PCGs, tRNA and rRNA genes) was 64,483 bp, accounting for 8.25% of the whole *P. simonii* mitogenome, while more than 90% of the mitogenome belonged to intergenic regions. The physical locations, gene length, and functional types of annotated genes were shown in Supplementary Table S4 and Figure 2. In the *P. simonii* mitogenome, most of the PCGs use ATG as the start codon, while *mttB* and *rpl16* cannot determine their start codons. It is unknown whether RNA-editing will likely correct these undetermined start codons. All the PCGs used TAA, TAG or TGA as stop codons, suggesting that there is no RNA-editing event happened in the stop codons (Ye et al., 2017).

All three chromosomes of the *P. simonii* mitogenome had protein-coding capability, in which most of the PCGs were annotated on a single chromosome, while *nad1* and *nad5* were separated by two different chromosomes (Figure 2). The exon1 of *nad1* was located on mtChr2, while the other four exons were located on mtChr1. The exon3 of *nad5* was located on mtChr3, while the other four exons were located on mtChr2. The number of introns was reported variably in different land plants (Mower, 2020). In *P. simonii* mitogenome, 8 protein-coding genes were identified harboring 17 *cis*-spliced introns with 27,298 bp in length (Supplementary Table S4), including three genes contained one intron (*rpl2*, *ccmFc*, and *rps3*), two genes contained two introns (*nad1* and *nad5*), two genes contained three introns (*nad2* and *nad4*), and one gene (*nad7*) contained four introns.

### Analyses of Genomic Syntenic Regions and Rearrangements in *Populus*

**FIGURE 3:**
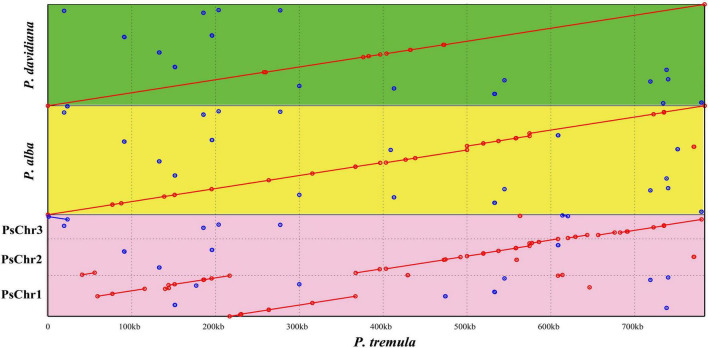
Mitogenomic syntenic analyses among four *Populus* species. The *P. tremula* mitogenome was set as reference. PsChr1, PsChr2, and PsChr3 represent the three circular chromosomes of *P. simonii* mitogenome. The green, yellow, and pink rectangular region represent the syntenic regions of *P. tremula* vs. *P. davidiana*, *P. tremula* vs. *P. alba*, and *P. tremula* vs. *P. simonii*, respectively. The red and blue lines refer direct and inverted syntenic regions, respectively.

Using the nucmer program of MUMmer v3.23, the mitochondrial evolution in *Populus* was investigated by detecting the syntenic regions and rearrangements between *P. tremula* mitogenome and other three mitogenomes, including *P. simonii*, *P. alba*, and *P. davidiana*. Between the mitogenomes of *P. tremula* and *P. simonii*, 56 local colinear blocks (LCBs) were detected accounting for 89% (695,519 bp) of the whole *P. simonii* mitogenome (Figure 3 and Supplementary Table S5). Between the mitogenomes of *P. tremula* and *P. alba*, 41 LCBs were detected accounting for 92.65% (776,825 bp) of the whole *P. alba* mitogenome. Between the mitogenomes of *P. tremula* and *P. davidiana*, 25 LCBs were detected accounting for 99.11% (772,408 bp) of the whole *P. davidiana* mitogenome. Since over 99% sequences of the *P. davidiana* mitogenome were co-linear with *P. tremula*, the differentiation of *P. tremula* and *P. davidiana* must be very recent. The dot plot further illustrated the sequence rearrangements in four mitogenomes. As clearly illustrated in Figure 3, there were only two large-scale rearrangements underwent between the mitogenomes of *P. tremula* and *P. davidiana*, at least 4 large-scale rearrangements occurred between the mitogenomes of *P. tremula* and *P. alba*, and at least 13 large-scale rearrangements occurred between the mitogenomes of *P. tremula* and *P. simonii*. The arrangements detected among four *Populus* mitogenomes did not disrupt the gene clusters, because the gene clusters were mostly located in syntenic regions.

### Analyses of Repeat Elements in *Populus simonii* Mitogenome

The variable genome size and structure of plant mitogenomes can be explained by variable repeat sequences, composing of SSRs, tandem repeats, and dispersed repeats. SSRs, also known as microsatellite DNA, which are DNA fragments consisting of tandem-repeated short units of 1–6 base pairs in length that are very useful molecular markers in identifying genetic diversity and unknown species. Using the online Microsatellite identification tool, 708 SSRs were identified in *P. simonii* mitogenome, including 296 mono-, 273 di-, 29 tri-, 89 tetra-, 16 penta-, and 5 hexa-nucleotide repeats (Supplementary Table S6). The total number of SSRs of *P. simonii* mitogenome was similar to those of other three *Populus* mitogenomes, but a little higher than those of other plants in Malpighiales, probably due to the different genome sizes. The repeat units of A/T, AG/CT, and AAAG/GTTT were found to be more prevalent than other repeat types in mononucleotide, dinucleotides, and tetranucleotides, respectively. Tandem repeats, also known as minisatellite DNA, are defined classically as tandem repeats of larger than 10 bp in length. They are widely distributed in eukaryotic genome and in some prokaryotes (Cheng et al., 2021). Using the online tool Tandem Repeats Finder, 7, 5, and 2 tandem repeats with lengths ranging from 13 bp to 33 bp in mtChr1, mtChr2, and mtChr3 were detected, respectively (Supplementary Table S7). Except for one tandem repeat located in coding region (*rrnL*), the others were all distributed in the intergenic spacers.

Besides SSRs and tandem repeats, a total of 322 dispersed repeats with lengths > 30 bp were detected in the *P. simonii* mitogenome using REPuter software (Table 3). The total length of dispersed repeats was 32,635 bp, accounting for 4.18% of the whole mitogenome. As shown Table 3, most of the repeats were 30 to 49 bp long (227 repeats, 70.5%), and the longest repeat was only 297 bp, with no repeats longer than 300 bp. Large repeats play crucial roles in genomic structure changes, and pairwise direct and inverted large repeats (>500 bp) may produce subgenomic or isomeric conformations (Bi et al., 2020). Since there were no repeats longer than 300 bp in the *P. simonii* mitogenome, no other subgenomic or isomeric conformations were detected. Due to the lack of large repeats in *Populus* mitogenomes (*P. simonii*, *P. alba*, *P. davidiana*, and *P. tremula*), the sizes and gene numbers of the four *Populus* mitogenomes were very conserved, and no multi-copy protein-coding genes in *Populus* mitogenomes were identified. By contrast, we identified 7, 4, 2 and 1 large repeats in *Passiflora edulis* (*Pa. edulis*), *Manihot esculenta* (*M. esculenta*), *Ricinus communis* (*R. communis*), and *Salix suchowensis* (*S. suchowensis*) mitogenomes, respectively. The *Pa. edulis* mitogenome contained the most repetitive sequences (total repeat length: 202,676 bp, 29.78% of the whole mitogenome), followed by *M. esculenta* (60,294 bp, and 8.83%) and *S. suchowensis* (50,339 bp, 7.81%).

**TABLE 3:** Comparison of dispersed repeats in nine Malpighiales plant mitogenomes.

Species	Repeat size (bp)							Longest repeat (bp)	Total repeat length (bp)	Proportion of genome (%)
	30–49	50–69	70–99	100–149	150–199	200–499	>500			
*P. simonii*	227	49	24	11	4	7	0	297	32,635	4.18
*P. alba*	242	43	30	19	4	6	0	308	35,485	4.23
*P. davidiana*	219	36	27	15	6	5	0	286	31,665	4.06
*P. tremula*	217	40	26	15	7	5	0	308	32,056	4.09
*S. suchowensis*	140	30	14	3	5	2	1	15,592	50,339	7.81
*S. brachista*	134	30	13	9	2	5	0	392	20,555	3.38
*M. esculenta*	189	52	25	22	9	9	4	32,495	60,294	8.83
*P. edulis*	177	53	37	20	13	17	7	4,930	202,676	29.78
*R. communis*	116	24	24	17	3	3	2	4,289	31,114	6.19

### Phylogenetic Analyses

**FIGURE 4:**
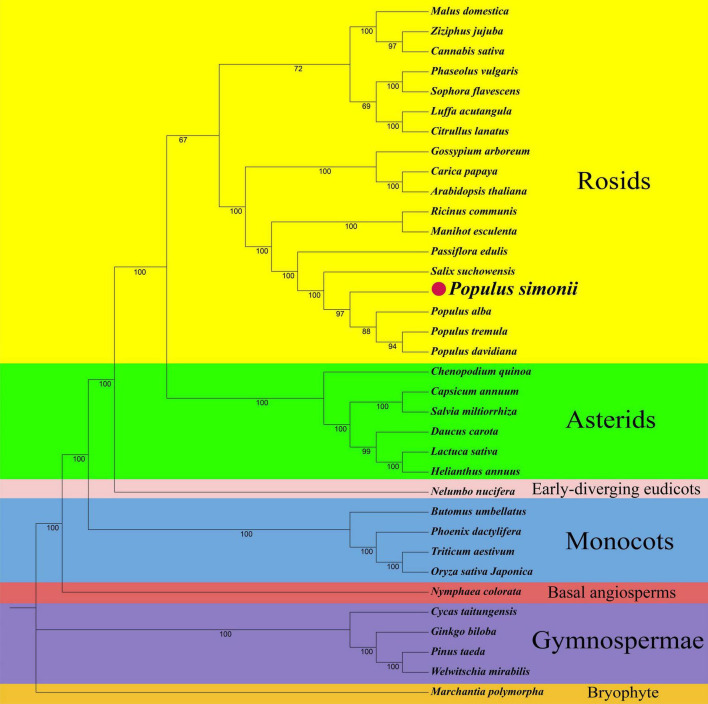
The phylogenetic relationships of *P. simonii* with other 34 represented land plants. Numbers on each node are bootstrap support values. Colors indicate the families of each species.

Phylogenetic analyses were performed based on the conserved PCGs of *P. simonii* and other 34 land plants, including 18 rosids, 6 asterids, one early-diverging eudicot of *Nelumbo nucifera*, four monocots, one basal angiosperm of *Nymphaea colorata*, 4 gymnosperms, and one plant (*Marchantia polymorpha*) in bryophyte. The abbreviations and GenBank accession numbers of all observed plants were listed in Supplementary Table S1. The maximum-likelihood (ML) tree strongly supported the separation of *P. simonii* and other three *Populus* plants with 97% bootstrap value, as well as the separation of rosids and asterids, the separation of eudicots and monocots (100%), and the separation of angiosperms and gymnosperms (100%) (Figure 4). The topology was highly similar to the APG IV system (Group Angiosperm Phylogeny, 2016).

### Frequent Protein-Coding Genes Loss and Acquisition in Land Plant Mitogenomes

The land plant mitogenomes have functionally diverse repertoires of 43 protein-coding genes, which is likely to be the full set of PCGs in the common ancestor of land plant mitogenomes. The plants in liverwort and moss have retained nearly all of the ancestral repertoire, while hornworts and some angiosperms have lost one-third or more of these ancestral genes (Supplementary Table S8). Strikingly, four mitochondrial genes involving the cytochrome c maturation (*ccm*) pathway have been retained in most land plants but lost in hornworts and ferns during land plant evolution. The genes encoding ATP synthase (*atp1*, *atp4*, *atp6*, *atp8*, and *atp9*), ubiquinol cytochrome c reductase (*cob*), cytochrome c oxidase (*cox1*, *cox2*, and *cox3*), and NADH dehydrogenase (*nad1*, *nad2*, *nad3*, *nad4*, *nad4L*, *nad5*, *nad6*, *nad7*, and *nad9*) were largely conserved among all land plant mitogenomes. The *rpl6* and *rps8* genes have been lost from all seed plants during the evolution, while *rps2* and *rps11* have been lost from most eudicot plants after the differentiation of eudicots and monocots. Significantly, the *rps14* gene was conserved in *P. alba*, *P. tremula*, and *P. davidiana*, but lost from the *P. simonii* mitogenome. To evaluate the absence pattern of *rps14* gene during the evolution of *P. simonii*, the *rps14* genes of *P. alba*, *P. tremula* and *P. davidiana* mitogenomes were used as query to search for homologous sequences in its chloroplast (NC_037418.1) and nuclear genomes (GCA_007827005.2) using BlastN. However, homologous sequences were not found in cp and nuclear genomes, suggesting that *rps14* gene was completely lost from *P. simonii* mitogenome during the divergence of *P. simonii* and other plants in the genus *Populus.*

## Discussion

### Mitochondrial Genome Size Variation

After several years of sequencing and study, it is now very clear that plant mitogenome is an evolutionarily dynamic entity exhibiting incredible diversity across plants in terms of size, gene content, and genome structure (Petersen et al., 2020). The primary explanations for the size variations in plant mitogenomes are the proliferation of repeat elements, the incorporation of foreign sequences, and gain or loss of large intra-genetic segments (Wu Z. Q. et al., 2020). Previous study of several mitogenomes of *Zea mays* found their size varied from 536 to 740 kb with large repeat elements (0.5–120 kb) accounting for most of the size variations (Allen et al., 2007). Another study estimated that approximately 20% of the mitogenome of *Malus domestica* was imported from other cellular compartments, including nucleus and chloroplast (Goremykin et al., 2012). Additionally, the mitogenome *A. trichopoda* was found to contain a large number of sequences transferred horizontally from green algae, mosses and other angiosperms (Rice et al., 2013).

In genus *Populus*, the mitogenome sizes are very conserved (Table 2), ranging from 779 kb (*P. davidiana*) to 838 kb (*P. alba*). Comparative repeat analyses of four *Populus* mitogenomes (Table 3) showed that there were no repeats larger than 350 bp in *Populus* mitogenomes, probably contributing to the stability of genome sizes, gene contents, and genome structures in the genus *Populus*. The extremely complex repeat patterns should be responsible for the various mitogenome sizes in plant species, however, mitogenome size is by no means only determined by repeats. The frequent transfers of foreign DNA among chloroplast, mitochondrion and nuclear can also contribute to the variable mitogenome sizes (Bergthorsson et al., 2003).

### Variation in Protein-Coding and tRNA Genes Among Plant Mitogenomes

Mitochondrial PCGs are largely conserved among liverworts and mosses, but vary substantially among and within land plants due to frequent losses and acquisitions of genes encoding ribosomal proteins and, to a lesser extent, genes involved in cytochrome c maturation and oxidative phosphorylation (Mower, 2020). With rare exception, most lineages of observed plants have retained between 30 and 41 (*Marchantia polymorpha* and *Scapania ornithopodioides*) PCGs in their mitogenomes, with functions ranging from oxidative phosphorylation to the maturation, translation, and transport of proteins (Burger et al., 2012). As shown in Supplementary Table S8, the genes encoding ribosome proteins (*rpl*- and *rps*- genes) and succinate dehydrogenases (*sdh3* and *sdh4*) among the observed 44 plant mitogenomes were particularly prone to lose from plant mitogenomes, whereas genes encoding oxidative phosphorylation subunits were much more likely to be retained during the angiosperm evolution (Adams, 2003; Park et al., 2015; Bi et al., 2016; Zhao et al., 2018).

**FIGURE 5:**
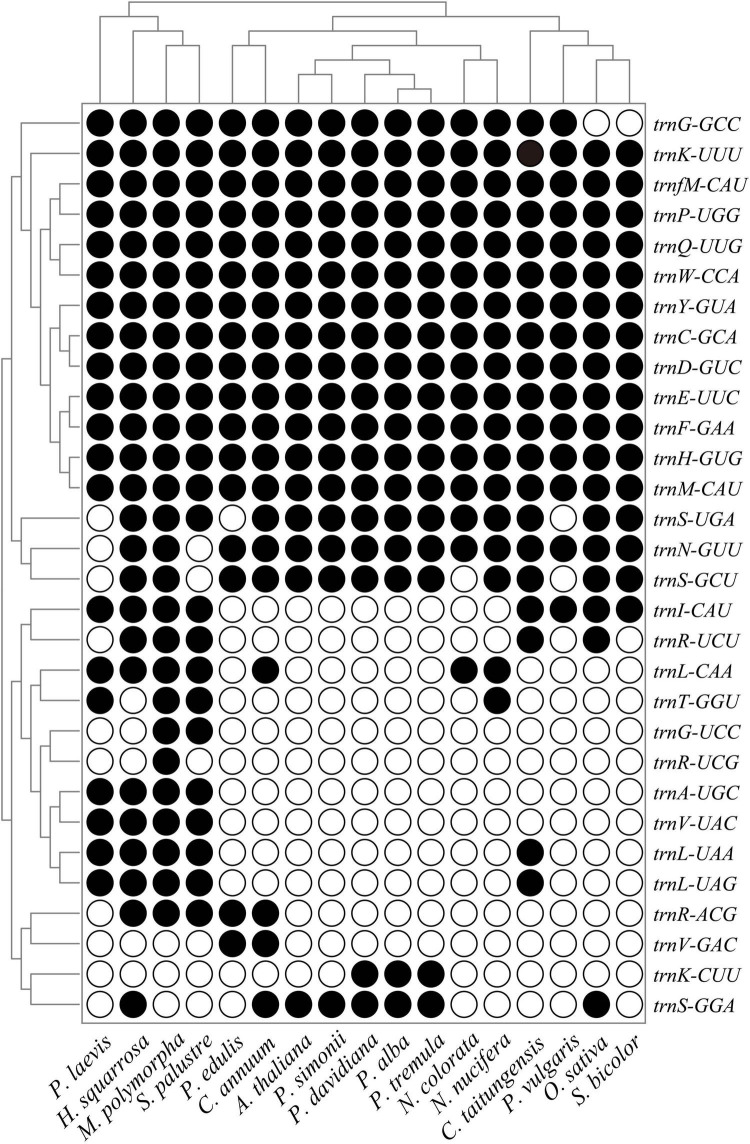
Mitochondrial tRNA gene contents among 17 representative plants. Black circle, gene is present; White circle, gene is absent. Species names are abbreviated as shown in Supplementary Table S1.

The loss and acquisition patterns of tRNA genes in land plants were also investigated in this study. Twelve tRNA genes (*trnC-GCA*, *trnD-GUC*, *trnE-UUC*, *trnF-GAA*, *trnH-GUG*, *trnK-UUU*, *trnM-CAU*, *trnfM-CAU*, *trnP-UGG*, *trnQ-UUG*, *trnW-CCA*, and *trnY-GUA*) were very conserved during the land plant evolution, while the other tRNA genes were prone to loss from one or more plant mitogenomes (Figure 5). In contrast to other plant mitogenomes in *Populus*, the *rps14* and *trnK-CUU* were lost from *P. simonii*, where *trnK-CUU* was transferred into its nuclear genome, and *rps14* was complete lost from *P. simonii* mitogenome during evolution. These lost genes can experience different fates; they may be completely lost from the cell (Adams, 2003), or functionally substituted by the same functional genes from plastids, nuclei, or the cytosol (Lei et al., 2013; Wang et al., 2021). Previous studies have reported that most mitochondrial lost tRNA genes were generally compensated by the corresponding cp-derived tRNA genes, such as *trnH-GUG*, *trnN-GUU*, *trnS-GGA*, and *trnW-CCA* (Bi et al., 2016; Wang et al., 2021). Additionally, some lost genes (*rps2*, *rps11*, and *rps19*) were transferred to the nucleus during evolution, which was the main pathway for PCGs loss in plant mitogenomes (Adams et al., 2002; Park et al., 2015). Frequent intracellular gene transfers among nucleus, chloroplast and mitochondrion promoted the movement of genetic material in organisms, and enabled the nucleus to control the organelle by encoding organelle-destined proteins and tRNA genes (Woodson and Chory, 2008).

### Multi-Chromosomal Structure in Plant Mitogenomes

Plant mitogenomes are conventionally depicted as circular molecules (Kozik et al., 2019), however, the increased availability of plant mitogenomes have revealed that the *in vivo* structure of plant mitogenome is far more complex than a single circular molecule would suggest (Sloan, 2013). The multi-chromosomal mitogenomes have been detected in many species, including ferns, gymnosperms, basal angiosperms, eudicots, and monocots. The mitogenome of the whisk fern *Psilotum nudum* (genome size: 629 kb) was assembled into two circular chromosomes (Guo et al., 2017). The largest mitogenomes sequenced to date are mainly from Gymnosperm species, but the number of chromosomes cannot be resolved accurately at his time due to their incomplete assemblies (Nystedt et al., 2013; Jackman et al., 2015). The mitogenome of the basal angiosperm *A. trichopoda* (genome size: ∼3900 kb) was detected to consist of five circular chromosomes ranging from 118.7 to 3179.3 kb (Rice et al., 2013). The number of chromosomes varies widely among eudicots, with the largest being the mitogenome of *Silene conica* (>128 chromosomes), while the number of chromosomes is concentrated between 2 and 5 in other multi-chromosomal eudicots (Wu, Z. Q. et al., 2020), such as *Actinidia chinensis* (Actinidiaceae) (Wang et al., 2019), *C. sativus* (Cucurbitaceae) (Alverson et al., 2011), *Camellia sinensis* (Ericaceae) (Zhang et al., 2019), *Fallopia multiflora* (Ericaceae) (Kim and Kim, 2018), *Solanum tuberosum* (Solanaceae) (Varre et al., 2019), and so on. For monocots, the number of multi-chromosomes were found to contain 2 or 3 chromosomes, while the mitogenome of *Gastrodia elata* (Orchidaceae) was assembled into 19 chromosomes (Yuan et al., 2018).

Unlike the fungal and animal multi-chromosomal mitogenomes in which the functional genes are distributed evenly between chromosomes, many chromosomes in plant multi-chromosomal mitogenomes do not contain any functional genes. For example, 20 out of 59 chromosomes in the *S. noctiflora* mitogenome were found to contain no functional genes (Wu et al., 2015a). All functional genes of the *C. sativus* mitogenome were found in the large chromosome, while none was detected in the other two small chromosomes (Alverson et al., 2011). In this study, we found that all three chromosomes of the *P. simonii* mitogenomes contain functional genes, and two protein-coding genes (*nad1* and *nad5*) were detected to be separated by two different chromosomes. Further transcriptome studies are needed to verify whether these two genes can be properly expressed in plant mitochondria.

### The Prospects of Plant Mitogenome Research

Because of the incorporation of foreign DNA, the proliferation of repeat elements, and the frequent gain or loss of intragenomic segments, the number of complete plant mitogenomes is far fewer than that of chloroplast genomes and animal mitogenomes. With the limited number of plant mitogenomes, it is difficult to investigate the dominant pattern of variation in multi-chromosomal mitogenomes at both inter- and intraspecific levels. Wu and Sloan predicted that the primary pattern of the multi-chromosomal variations should be the chromosome loss events after large ancestral expansions (Wu and Sloan, 2019), however, they could not get the conclusive result only from the limited genomic data. In this study, the *P. simonii* mitogenome was assembled into three circular chromosomes, with an atypical multi-chromosomal mitogenome rather than the typical single circular structure in other sequenced *Populus* mitogenomes. Therefore, *Populus* can be stand as a superb model genus to explore the evolutionary pattern and the drivers for the variation of multi-chromosomal structures. Many questions remain about the molecular factors and mechanisms of the variation of multi-chromosomal structures in plants, for example, what potentially novel mechanisms regulate and control the replication and segregation of multi-chromosomal mitogenomes during cell division? How do they achieve fusions of the inner and outer membranes? Why these “empty” mitochondrial chromosomes retained, and what purpose do they play at different stages of development? In the near future, RNA-seq, small RNA-seq and multigenerational studies involving controlled crosses may shed light on these questions.

Plant mitochondria have many features that distinguish them from fungal and animal mitochondria, such as abundance, smallness, fast movement, and roles in cytoplasmic male sterility (Arimura, 2018). These plant-specific features provide many challenging and exciting opportunities for future research. With the continuing development of long-read sequencing method, such as PacBio and Oxford Nanopore sequencing, more and more accurate mitogenome structures can be acquired in the near future, which are imperative for better elucidating their biological functions, replication and recombination mechanisms, and their unique evolutionary trajectories (Kozik et al., 2019).

## Conclusion

In this study, we assembled the multi-circular mitogenome of *P. simonii*, which was reported in genus *Populus* for the first time. Whole-genome alignment analyses found that the four *Populus* mitogenomes shared more than 90% local colinear blocks, indicating the fission of poplar mitogenome in *P. simonii* must be very recent. There were no repeats longer than 350 bp in four *Populus* mitogenomes, probably contributing to the stability of genome sizes and gene contents in genus *Populus*. The gene loss of *rps14* gene and horizontal transfer of *trnK-UUU* gene need to be studied to elucidate their specific mechanisms in the future. Further phylogenetic analyses based on *P. simonii* and other 34 plant mitogenomes provide new clues for phylogenetic relationships, especially for Salicaceae and Malpighiales. In summary, as the first reported multi-circular mitogenome in *Populus*, this study of *P. simonii* mitogenome will provide not only an important atypical mitogenome resource for the comparative and functional genomic research in *Populus*, but also a reliable clue for the research of their unique evolutionary trajectories.

## Data Availability Statement

The PE and PacBio sequencing data of *P. simonii* have been deposited in the NCBI Sequence Read Archive (SRA) repository under SRR3204721 and SRR9887262, respectively. The multi-circular mitogenome of *P. simonii* is available in NCBI Nucleotide Database under the GenBank accessions: MZ905370, MZ905371, and MZ905372. All the other mitogenome sequences used in this study are available in the NCBI Genome Database (https://www.ncbi.nlm.nih.gov/genome/organelle/) under the GenBank accessions listed in Supplementary Table 1.

## Author Contributions

CB: conceptualization, methodology, investigation, software, visualization, writing of original draft, writing of review and editing. YQ: data curation, resources, and software. JH: validation and funding acquisition. KW: validation and formal analysis. NY: writing of review and editing, methodology, and software. TY: conceptualization, funding acquisition, supervision, writing of review and editing. All authors: contributed to the article and approved the submitted version.

## Conflict of Interest

The authors declare that the research was conducted in the absence of any commercial or financial relationships that could be construed as a potential conflict of interest.

## Publisher’s Note

All claims expressed in this article are solely those of the authors and do not necessarily represent those of their affiliated organizations, or those of the publisher, the editors and the reviewers. Any product that may be evaluated in this article, or claim that may be made by its manufacturer, is not guaranteed or endorsed by the publisher.

## Footnotes

1
https://www.ncbi.nlm.nih.gov/genome/organelle/

2
https://www.pacb.com/support/software-downloads

3
http://www.vcru.wisc.edu/simonlab/sdata/software/

4
https://github.com/lbcb-sci/racon

5
http://dogma.ccbb.utexas.edu/mitofy/

6
https://chlorobox.mpimp-golm.mpg.de/geseq.html

7
https://webblast.ipk-gatersleben.de/misa/index.php

8
https://tandem.bu.edu/trf/trf.html

9
https://bibiserv.cebitec.uni-bielefeld.de/reputer

10
https://github.com/bichangwei/Populus-simonii-mitogenome

## Supplementary Material

Supplementary Figure S1 The distribution of contig length and read depth in the de novo assembly by Newbler v3.0.

Supplementary Figure S2 Comparison of *P. tremula* and *P. simonii* mitochondrial genomes. PsChr1, PsChr2, and PsChr3 represent the three circular molecules of *P. simonii* mitogenome, respectively.
