# Supplementary figures and images for: Deciphering the Multi-Chromosomal Mitochondrial Genome of Populus simonii
Source: Front Plant Sci. 2022 Jun 15;13:914635. doi: 10.3389/fpls.2022.914635 (PMC9240471; doi:10.3389/fpls.2022.914635)

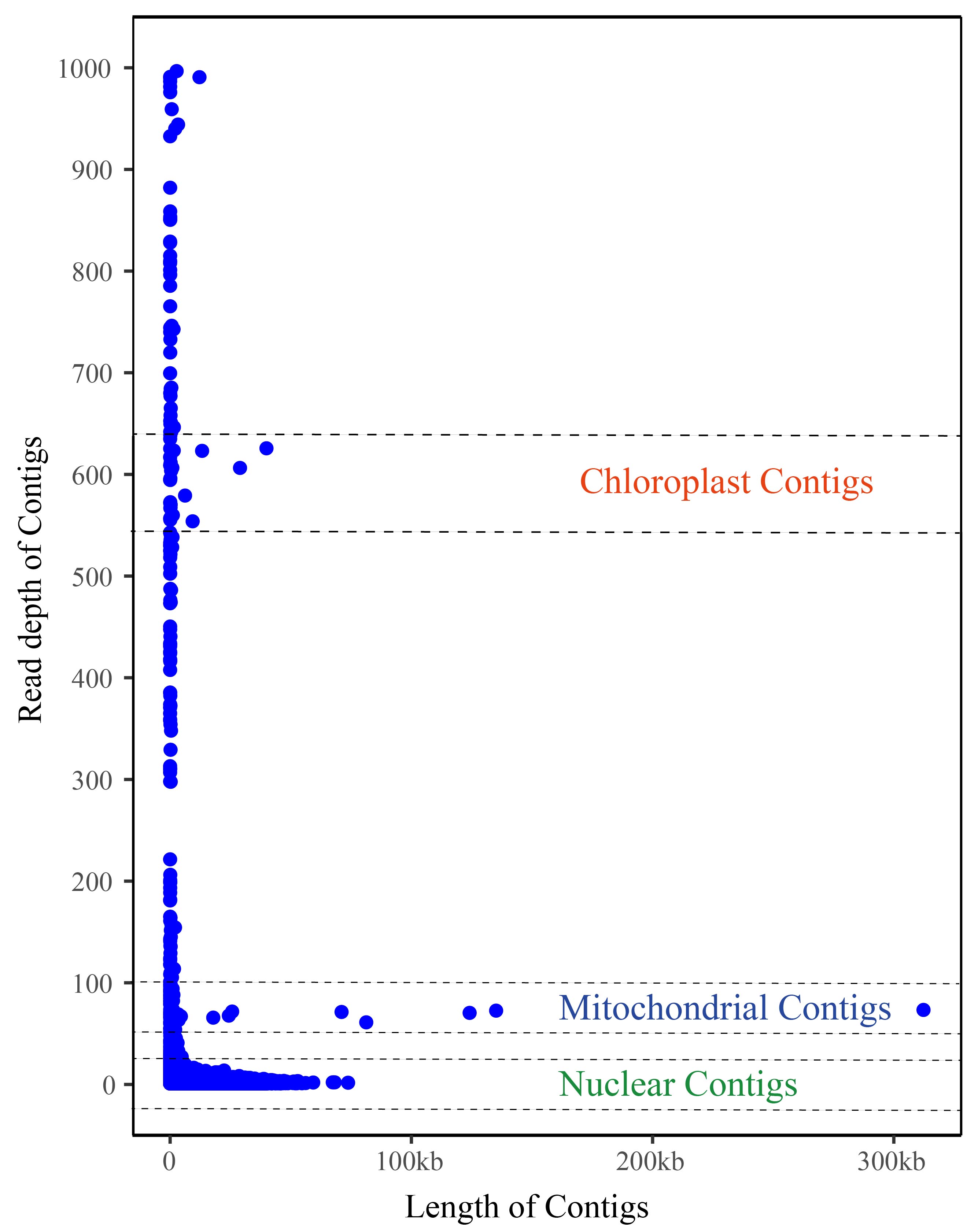

Supplement: Supplementary Figure S1 — The distribution of contig length and read depth in the de novo assembly by Newbler v3.0. [file Image_1.JPEG]

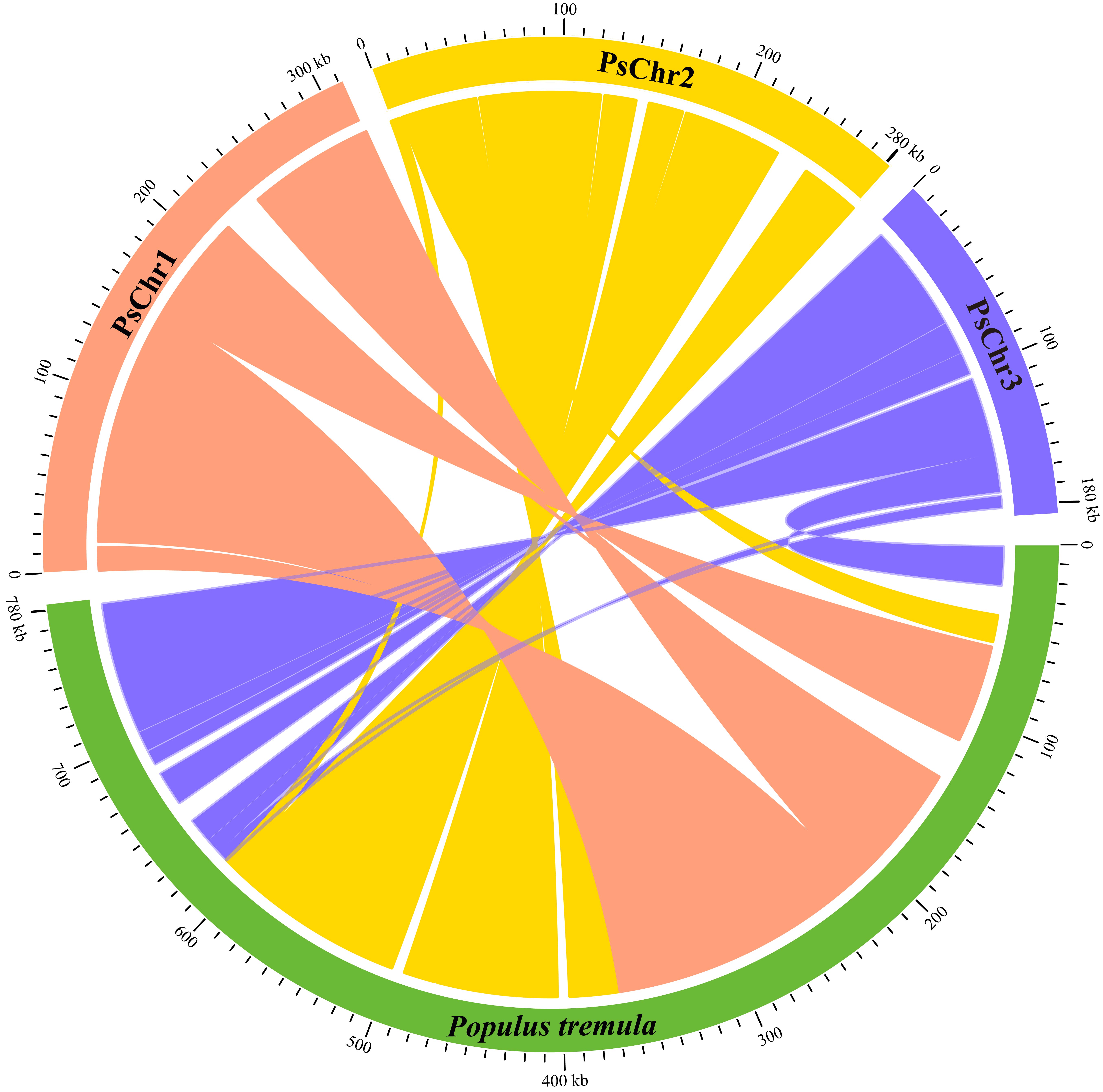

Supplement: Supplementary Figure S2 — Comparison of P. tremula and P. simonii mitochondrial genomes. PsChr1, PsChr2, and PsChr3 represent the three circular molecules of P. simonii mitogenome, respectively. [file Image_2.JPEG]
